# Dairyman Held Responsible for Typhoid Germs in Milk

**Published:** 1905-02

**Authors:** 


### DAIRYMAN HELD RESPONSIBLE FOR TYPHOID GERMS IN MILK.

A verdict given against the Aylesbury Dairy Company, one of the largest firms engaged in the sale of milk in London, is of far-reaching importance, applying, as it does, an old principle in law to a new set of circumstances. In July last year there was an epidemic of typhoid fever in Ealing, a London suburb. Among the victims was a woman who died. She had partaken of milk supplied by the Aylesbury Dairy Company. Her husband, believing that the milk was the source of the disease, sued the company for damages. His counsel pointed out that the company, in their advertisements, guaranteed that their milk was free from disease germs. He also proved that the part of the company's supply was drawn from a farm where there had been a case of typhoid fever. If the milk had been polluted then he contended that there had been a breach of warranty. There was some difference in the expert evidence as to the contamination of the milk. Dr. Tresh, examiner in state medicine at the University of London, said that there was a suspicion as to the milk, but the proof was far from conclusive. Evidence was given that in June, July and August, 1903, 21 cases of typhoid fever occurred in this suburb and in 12 the milk was supplied by the defendants. For the defense it was shown that elaborate precautions were taken by the company to insure the purity of the milk. The judge instructed the jury that if a purveyor sold food injurious to health he was liable. The jury found a verdict for $530, the amount of expense to which the woman's husband had been put. It is thought by health officers that the decision will do much good by making dairy companies more particular.—*Journal American Medical Association.*

